# There Is No Association Between the Number of Stent Retriever Passes and the Incidence of Hemorrhagic Transformation for Patients Undergoing Mechanical Thrombectomy

**DOI:** 10.3389/fneur.2019.00818

**Published:** 2019-08-08

**Authors:** Ameer E. Hassan, Hari Kotta, Umar Shariff, Laurie Preston, Wondwossen Tekle, Adnan Qureshi

**Affiliations:** ^1^Department of Neurology and Radiology, University of Texas Rio Grande Valley, Harlingen, TX, United States; ^2^Department of Neurology, University of Texas Medical Branch, Galveston, TX, United States; ^3^Department of Neurology, University of Missouri, Columbia, MO, United States

**Keywords:** thrombectomy, hemorrhagic transformation (HT), stent retriever, endovascular, intracranial hemorrhage

## Abstract

**Background:** Previous research has focused on the association between hemorrhagic transformation (HT) incidence and pre-procedural variables (i.e., baseline variables) rather than the association between HT incidence and endovascular treatment (EVT) procedural variables (e.g., stent retriever passes).

**Objective:** To assess the association, if any, that exists between the number of stent retriever passes per procedure and the incidence of HT for patients undergoing mechanical thrombectomy.

**Methods:** An endovascular database from a comprehensive stroke center was used to collect data on EVT patients treated with Trevo, Solitaire, or Penumbra stent retrievers from the years 2012 to 2017. Statistical analyses were conducted on the stent retriever passes, demographics, morbidities, medication usage, and outcomes and their association with HT.

**Results:** Of the 329 total patients, 46 (14%) had HT. The HT group had an average [SD] of 1.65 [0.67] and range of [1–3] passes per procedure while the non-HT group had an average [SD] of 1.63 [0.86] and range of [1–5] passes per procedure. Admission NIHSS score (*p* = 0.0003) and the incidence of diabetes mellitus (DM) (*p* = 0.05) were significantly higher in the HT group. Subdividing HT into symptomatic and asymptomatic ICH groups failed to display significant differences in the distribution of the stent retriever passes (*p* = 0.969). The number of passes failed to show any association with HT (*p* = 0.804) while admission NIHSS score was found to have an OR of 1.07 (95%CI: 1.029–1.121, *p* = 0.001) with HT incidence.

**Conclusion:** No significant association was found between HT incidence and the stent retriever passes. Further multicenter studies are warranted to corroborate our results.

## Introduction

Hemorrhagic transformation (HT) presents as a prolific complication to any patient undergoing reperfusion therapies such as IV thrombolysis and/or endovascular treatment (EVT). HT, particularly the parenchymal hematoma version 2(PH-2), significantly increases the risk of subsequent neurological deterioration and mortality (1). While several HT subtypes have been documented to be more clinically relevant than others (i.e., HT-1, HT-2, etc.), our study was primarily a safety outcomes analysis between the overall incidence of HT and the number of stent retriever passes.

Previous research has identified several pre-procedural risk factors of HT for acute ischemic stroke patients including pre-existing morbidities such as “atrial fibrillation (AF), diabetes mellitus (DM), and congestive heart failure (CHF)” (2–4), radiographic biomarkers such as “relative CBV values, relative CBF values, and collateral flow” (5), laboratory values such as “platelet count, glucose levels, and levels of total cholesterol” (2) and clinical variables such as admission NIHSS score (6). Several studies have utilized such pre-procedural variables to create strong predictive models for the incidence of HT. For example, Kalinin et al. created the Hemorrhagic Transformation Index (HTI) to predict HT incidence within 14 days for acute ischemic stroke patients using the Alberta Stroke Program Early CT (ASPECTS) score, NIHSS score, hyperdense MCA sign, and incidence of Atrial Fibrillation upon admission (6).

Despite recently proliferating research, the impact of procedural factors such as stent retriever passes on HT has not been well-established in the era of stent-retrievers. Current stent retriever manufacturers recommend a maximum of 2 passes per device and not more than 3 passes per vessel as per the IFU (Medtronic, Irvine, CA).

Our aim with this paper is to elucidate a potential association, if any, between the number of passes with a stent retriever and the incidence of HT. This study is to be regarded primarily as a safety outcomes analysis, although clinical outcomes are discussed extensively as well.

## Methodology

All acute ischemic stroke patients who underwent EVT with a stent retriever were selected from a prospectively maintained endovascular database of a comprehensive stroke center from 2012 to 2017. A modified “Solumbr” approach was utilized in which a guide catheter, a distal access catheter and a microcatheter were used to cross the lesion. A stent retriever was subsequently deployed for 5–7 min and pulled back slightly under aspiration to cork it into the distal access catheter, and then both were removed together slowly while under constant aspiration via a pump. Heparin is not administered in any of our stroke cases; however, 2,000 units/bag are connected to the flush lines. GP2b/3a inhibitors are not routinely used. EVT is typically terminated after 60 min of intervention time (7, 8). Baseline clinical characteristics, endovascular procedural variables, safety outcome rates, and patient outcomes were gathered from the selected patients. Baseline characteristics included patient demographic data, pre-existing morbidities, medication usage, pre-procedural mTICI score, admission NIHSS score, occlusion location, and admission modified Rankin Scale (mRS) score (Table 1). Endovascular procedural variables included the number of passes per operation with a stent retriever, time from stroke onset to arrival at hospital, time from emergency department (ED) arrival to time of recanalization, and time from groin puncture to time of successful recanalization (defined as mTICI 2b-3), stent-retriever sizing, and catheter type. Safety outcome data included incidence of HT, mass effect, and intracranial hemorrhage (ICH). HT was defined as any ICH in the territory of the initial ischemic event during hospitalization and confirmed by multiple non-contrast head CT scans or MRIs of the brain according to radiology. HT incidence was further subdivided into asymptomatic ICH and symptomatic ICH. Symptomatic ICH was defined as a neurological deterioration of 4+ points in the NIHSS scale score within 24 h post-procedure. Patients who received mechanical thrombectomy using Trevo, Solitaire, or Penumbra stent retrievers were dichotomized according to the incidence of HT. Patient outcomes were assessed by analyzing post-procedure NIHSS and mRS scores as well as mortality rate. NIHSS scores were collected upon admission, 24 h post-procedure, and time of discharge. The mRS scores were specifically used to assess functional independence and were collected upon admission, time of discharge, as well as at 90 day follow ups. Patients with an mRS score between 0 and 2 were deemed functionally independent at the time of collection. The study and protocol were approved by the local Metro West Institutional Review Board.

**Table 1:** Results from the univariate statistical analyses performed on baseline variables and endovascular procedural variables between HT(+) and HT(–) patients.

**Baseline variables**	**HT(+) (46)**	**HT(–) (283)**	***p*-value**
Age (mean) [SD]	71.9 [11.17]	70.7 [13.2]	0.527
**Ethnicity**			
African (%)	0 (0%)	1 (0.35%)	
Hispanic (%)	35 (76.1%)	206 (72.8%)	
White (%)	11 (23.9%)	74 (26.1%)	
Other (%)	0 (0%)	2 (0.71%)	0.889
**Sex**			
Male (%)	23 (50.0%)	149 (52.7%)	
Female (%)	23 (50.0%)	134 (47.3%)	0.7529
**NIHSS** score upon admission (median) [range]	20.59 [6.25]	16.05 [7.97]	0.00027
**Morbidities**			
Hypertension (%)	41 (89.1%)	250 (88.3%)	0.99
CHF (%)	2 (4.3%)	32 (11.3%)	0.195
CAD (%)	13 (28.3%)	88 (31.1%)	0.8633
Hyperlipidemia (%)	24 (52.2%)	163 (57.6%)	0.5233
Currently smoking (%)	2 (4.3%)	29 (10.2%)	0.2804
Diabetes mellitus (%)	27 (58.7%)	121 (42.8%)	0.0548
History of stroke/TIA (%)	10 (21.7%)	63 (22.3%)	0.99
Pre-treatment IV tPA (%)	22 (47.8%)	113 (39.8%)	0.334
**Pre-procedure mTICI score**			
0 (%)	41 (89.10%)	239 (83.0%)	
1 (%)	2 (4.35%)	26 (9.19%)	
2a (%)	3 (6.52%)	18 (6.0%)	0.551
**Occlusion location**			
MCA	29 (63.04%)	192 (67.77%)	
Carotid	13 (30.43%)	66 (23.30%)	
M2	2 (4.35%)	9 (3.18%)	
PCA, VA, ACA	2 (4.35%)	16 (5.65%)	0.844
Tandem occlusion	8 (17.4%)	79 (27.9%)	0.133
Procedural variables			
**Number of passes per procedure**			
1	21 (45.7%)	156 (55.1%)	
2	20 (43.5%)	84 (29.7%)	
3	5 (10.9%)	31 (11.0%)	
4	0 (0%)	9 (3.2%)	
5	0 (0%)	2 (0.71%)	0.201
**Time from:**			
Onset to arrival (hours) [*SD*]	4.02 [4.21]	4.39 [4.00]	0.626
ED arrival to recanalization (minutes) [*SD*]	53.50 [15.37]	58.68 [29.4]	0.245
Groin puncture to recanalization (minutes) [*SD*]	37.13 [15.47]	41.57 [28.1]	0.297
Stent-retriever sizing^*^			
3x, 4x (%)	20 (50%)	123 (48.8%)	
6x (%)	20 (50%)	129 (51.2%)	0.889
**Catheter type**			
Guide catheter (%)	46 (100%)	275 (97.2%)	
Balloon catheter (%)	0 (0%)	8 (3.2%)	0.606

* *Size 5x not included in analysis*.

## Statistical Analysis

Patient data were dichotomized according to the incidence of HT and analyzed via univariate and multivariate analyses. To assess statistical significance between the collected variables and the incidence of HT, a 2-sample *t*-test was performed on the continuous variables while Fisher's Exact test was performed on the categorical variables. Statistically significant data (i.e., *p* < 0.1) were analyzed further. A multivariate logistical analysis was conducted in which all statistically significant variables were incorporated together in addition to the number of passes per procedure to develop odds ratios for the incidence of HT. All statistical analyses were conducted using IBM SPSS Statistics.

## Results

A total of 329 patients underwent EVT with a stent retriever between the years 2012 and 2017. Forty-six (14%) patients developed HT (HT+) while the remaining 283 (86%) did not (HT–). The average age for all patients was 71 ± 12.9 and the mean admission NIHSS score for all patients was 16.7 ± 7.9. Additionally, the mean time of onset to time of arrival at the hospital was 4.08 ± 4.17 h while mean time from ED arrival to recanalization was 57.95 ± 27.44 min, and mean time from groin puncture to recanalization was 40.95 ± 26.33 min. Recanalization was achieved in 297 (90%) of all patients while good clinical outcome (mRS 0–2 after 90 days) was achieved in 46% of all patients and the rate of mortality at 90 days was 28%.

Table 1 displays the results from the univariate statistical tests performed on the baseline variables and endovascular procedural variables between HT(+) and HT(–) patient groups. The HT(+) patients had a range of 1–3 passes per procedure while the HT(–) patients had a range of 1–5 passes per procedure. While the number of passes with a stent retriever for HT(+) patients held a median of 2 and a mean of 1.652 ± 0.67, the median for HT(–) patients held a median of 1 and a mean of 1.63 ± 0.86. Subsequent statistical analyses, namely the Fisher exact test (*p* = 0.201) and multivariate logistical analysis (OR: 1.023, *p* = 0.903, 95% CI: 0.903–1.023), displayed these differences to be statistically insignificant. Admission NIHSS score (*p* = 0.00027) and incidence of diabetes mellitus (DM) (*p* = 0.055) were both found to have statistically significant association with the rate of HT. The HT(+) group had an Intravenous tissue Plasminogen Activator (IV tPA) usage rate of 47.8% while the HT(–) group had an IV tPA rate of 39.8% (*p* = 0.334). None of the temporal procedural variables, namely the time from onset to arrival at hospital (*p* = 0.623), time from arrival at ED to time of recanalization (*p* = 0.245), or time from groin puncture to time of recanalization (*p* = 0.297), were found to have significant association with HT.

Table 2 displays the results from comparing the distribution of the number of passes between asymptomatic ICH and symptomatic ICH subdivisions of the HT (+) group. Our study found a remarkably similar distribution of the number of passes between both groups (*p* = 0.969).

**Table 2:** Results from comparing the distribution of the number of passes between symptomatic ICH and asymptomatic ICH groups.

**Number of passes**	**Symptomatic ICH (11)**	**Asymptomatic ICH (35)**	***p*-value**
1	5 (45.5%)	16 (45.7%)	
2	5 (45.5%)	15 (42.9%)	
3	1 (9.1%)	4 (11.4%)	0.969

Table 3 delineates the results from the univariate statistical tests performed on endovascular safety outcome rates and patient outcomes between HT(+) and HT(–) patient groups. Significant safety outcome data included higher rates of symptomatic ICH (*p* < 0.0001), asymptomatic ICH (*p* < 0.0001), as well as mass effect (*p* < 0.0001) for the HT(+) group. Significant patient outcome data included higher NIHSS 24-h score (*p* < 0.0001), NIHSS discharge score (*p* = 0.00012), mRS discharge score (*p* = 0.0005), mRS 90-day score (*p* = 0.0278), and incidence of death up to 90 days (*p* = 0.0044) for the HT(+) group. The significant admission NIHSS score, 24-h NIHSS score, and discharge NIHSS score data obtained warranted additional statistical testing to investigate the amount of change in NIHSS score between both groups [i.e., HT(+) vs. HT(–)], namely the intergroup difference in the change between NIHSS score at 24-h and admission NIHSS (*p* = 0.0521). We also looked at the intergroup difference in the change between admission and discharge NIHSS score (*p* = 0.0203). The HT(–) group had a 3.44 ± 8.8 decrease from admission NIHSS score to 24-h score and a 3.90 ± 13.1 decrease from admission to discharge NIHSS score while the HT(+) group had a decrease of 0.63 ± 8.22 points from admission to 24-h NIHSS score but an increase of 1.40 ± 15.42 points from admission to discharge NIHSS score. The large SD values are largely attributable to the presence of negative NIHSS difference values.

**Table 3:** Results from the univariate statistical tests performed on safety outcome rates and patient outcomes between HT(+) and HT(–) patient groups.

**Clinical outcomes**	**HT(+) (46)**	**HT(–) (283)**	***p*-value**
Recanalization (%)	41 (89.1%)	261 (92.2%)	0.478
mRS (0–2) upon discharge (%)	3 (6.5%)	84 (29.7%)	0.0005
mRS (0–2) upon 90 days (%)	10 (21.7%)	89 (31.4%)	0.0278
Death (%)	18 (39.1%)	57 (20.1%)	0.0044
NIHSS upon 24 h (median) [range]	21 [1–34]	12 [0–32]	<0.0001
NIHSS upon discharge (median) [range]	18 [1–42]	8 [0–42]	0.0002
**NIHSS DIFFERENCES**			
NIHSS upon 24 h—NIHSS upon admission (mean) [SD]	−0.63 [8.22]	−3.44 [8.8]	0.0521
NIHSS upon discharge—NIHSS upon admission (mean) [SD]	+1.40 [15.32]	−3.90 [13.1]	0.0203
**SAFETY OUTCOMES**			
Asymptomatic ICH (%)	35 (76.1%)	11 (3.9%)	<0.0001
Symptomatic ICH (%)	11 (23.9%)	8 (2.3%)	<0.0001
Mass effect (%)	15 (32.6%)	18 (6.4%)	<0.0001

Subsequently, a multivariate logistic regression was performed using the statistically significant variables as predictor variables and the incidence of HT as the dependent variable (Table 4). The only significant predictor variable for HT obtained from multivariate logistic regression however was NIHSS admission score, which held an OR of 1.074 (*p* = 0.001; 95% CI: 1.029, 1.121). The number of passes with a stent retriever variable (*p* = 0.804) showed no statistically significant impact upon the multivariate logistical regression of the incidence of HT.

**Table 4:** Results from multivariate regression analyses using NIHSS score upon admission and incidence of DM to predict the incidence of HT.

**Baseline variables with univariate *p* < 0.1**	**OR**	***p*-value**	**Lower 95% CI boundary**	**Upper 95% CI boundary**
NIHSS	1.074	0.001	1.029	1.121
^*^DM	1.85^*^	0.069^*^	0.954^*^	3.57^*^

* *Deemed insignificant because of p > 0.05 and 95% CI being both below and above 1*.

## Discussion

This study has shown that the number of passes with a stent retriever is not significantly associated with the incidence of HT. While the difference in the distribution of the number of passes per procedure between HT(+) and HT(–) groups was found to be insignificant (*p* = 0.201), a wider range of passes [1–5] was observed in the HT(–) group than that of the HT(+) group [1–3].

While admission NIHSS scores have historically held strong association with post-procedure functional independence rates, our study found that admission NIHSS score was likewise significantly associated with the incidence of HT within both univariate and multivariate analyses. Prevalence of DM was an additional baseline variable associated with the incidence of HT in our univariate analysis (*p* = 0.055). Jiang et al. found that the risk of symptomatic ICH in diabetic patients had an OR ≥ 7 in both univariate and multivariate models, hypothesizing that the substantial risk was in part due to the increased vascular aging typical of diabetic patients (9). Hyperglycemia, although not directly measured in our study, has also been associated with increased rates of reperfusion injury and lower rates of both recanalization (mTICI 2b-3) and functional independence post-procedure (10). The temporal procedural variables in our study, namely the time from stroke onset to time of hospital arrival, time from arrival at ED to time of recanalization, and time of groin puncture to time of recanalization failed to show any significant association with HT. Jiang et al. found that increased time between groin arterial access and recanalization was significantly associated with higher rates of symptomatic ICH (OR 1.01, 95% CI: 1.00–1.02) as well as functional outcome OR 2.97, 95% CI 1.00–8.83 (9). However, the average groin to recanalization time of 114.51 ± 63.65 (9) min reported by Jiang et al. was significantly greater than of the average time of 40.95 ± 26.33 min observed in our study.

The HT(+) patient group had significantly higher NIHSS scores at 24 h post-procedure (*p* = 0.0005) and at the time of discharge (*p* = 0.0002) compared to the HT(–) group. The HT(+) group was also found to have significantly lower proportions of patients with post-operative functional independence (mRS 0–2) at discharge (*p* = 0.0005) as well as at 90 days (*p* = 0.0278) compared to the HT(–) group. Arimura et al. likewise found that postoperative ICH incidence was highly associated with decreased rates of functional independence upon 90 days (*p* = 0.004) (11).

Subdividing HT into asymptomatic ICH and symptomatic ICH groups did not show any significant differences in the distribution of the number of stent retriever passes (*p* = 0.969). As such, our study seems to support the inference that the 4+ increase in NIHSS score indicative of symptomatic ICH was not significantly attributable to the number of stent retriever passes.

Another significant finding in our study was that the majority of ICH (70.8%) were asymptomatic. Gill et al. obtained similar results upon analyzing safety outcome rates of previous stent retriever trials (i.e., Solitaire, Trevo, and preSet trials), in which he found that 1–5% of all cases were documented with symptomatic ICH while 7–30% were documented with asymptomatic ICH (12). Procedural risk factors exclusive to EVT only such as mechanical perforation or dissection of the vessel wall, stent-retriever size, catheter type, improper visualization of artery via x-ray fluoroscopy, as well as lesions acquired through retrieving stent-devices have been posed as explanations for this aberrant increase in ICH frequency (13, 14).

## Limitations

This study was performed retrospectively and was not randomized. Although it is established that certain categories of HT exist (i.e., HT-1, HT-2, etc.) that are not as clinically salient as others, our study regards HT as a safety outcome and includes the overall incidence of HT. Furthermore, given that procedural variables have been found to be highly variable between institutions (e.g., the significantly different times between groin puncture and recanalization across institutions noted above) our findings may not be generalizable to a very large-scale context. Corroboratory evidence would consist of multicenter retrospective studies and/or studies with larger ranges of stent retriever passes per procedure investigating our claim.

## Conclusion

This study found the number of passes with a stent retriever to be insignificantly associated with the incidence of HT. Admission NIHSS score and to a lesser degree DM were found to be much more associated with the incidence of HT. While our study primarily focused on the number of stent retriever passes, continuing to delimit and investigate other procedural variables are imperative steps toward improving safety outcome rates and patient outcomes.

## Data Availability

Datasets are available on request.

## Author Contributions

AH provided research question, analyzed the data, and revised the paper. HK developed the statistical analyses, drafted the paper, and revised the paper. US monitored initial data abstraction. LP monitored development of database and navigated endovascular database. WT and AQ revised the paper.

### Conflict of Interest Statement

The authors declare that the research was conducted in the absence of any commercial or financial relationships that could be construed as a potential conflict of interest.
